# The fascinating history of papillary thyroid carcinoma nuclei: revelation of two nuclear morphologies—“Classical papillary” and “papillary‐like”, with different pathobiologic characteristics

**DOI:** 10.1002/cncy.70100

**Published:** 2026-04-22

**Authors:** N. Paul Ohori, Jeremy M. Minkowitz, Yuri E. Nikiforov, Raja R. Seethala

**Affiliations:** ^1^ Department of Pathology University of Pittsburgh and UPMC‐Presbyterian Pittsburgh Pennsylvania USA

**Keywords:** classification, follicular variant, history, molecular testing, papillary carcinoma, thyroid

## Abstract

The nucleus of papillary thyroid carcinoma (PTC) has had a fascinating history since its early descriptions by Lindsay in 1960 and his designation of follicular variant papillary thyroid carcinoma (FVPTC) as a subtype of follicular carcinoma (FC). Later, Chen and Rosai revived the awareness of FVPTC and aligned this neoplasm with PTC. From this era, PTC became a nuclear diagnosis and was expanded to include FVPTC, even when noninvasive and encapsulated. However, this practice was prone to interobserver variability. The main issue centered on the interpretation of two types of nuclei: the widely accepted “classical papillary nucleus” with well‐developed features such as nuclear pseudoinclusions and grooves and the other “papillary‐like nucleus” with subtle and delicate nuclear features. Subsequently, some individuals recognized papillary‐like nucleus in most follicular‐patterned neoplasms and diagnosed them as FVPTC, whereas others diagnosed them as follicular adenoma. In the 2000s, noninvasive, encapsulated FVPTC was recognized as an indolent entity, and later relabeled as noninvasive follicular thyroid neoplasm with papillary‐like nuclear features. Molecular analyses revealed two main families of drivers for PTC, *BRAF V600E* or *BRAF V600E*–like mutations, which aligned with classical papillary nucleus, papillary architecture, and conventional PTC outcomes, and *RAS* or *RAS*‐like mutations that were associated with papillary‐like nucleus, follicular architecture, and follicular‐patterned neoplasm outcomes. The story has come full circle, and the recent proposal to incorporate FVPTC into follicular adenoma is in keeping with Lindsay’s original concept. By the current understanding, these nuclei are classified better by molecular associations.

## Abstract 2

The nucleus of papillary thyroid carcinoma has had a fascinating history with eventual recognition of the widely accepted classical papillary nucleus (associated with *BRAF V600E* or *BRAF V600E*–like mutations) and the papillary‐like nucleus (associated with *RAS* or *RAS*‐like mutations) with subtle features. For invasive follicular–patterned neoplasms, the papillary‐like nucleus has shifted its association from follicular carcinoma to papillary thyroid carcinoma and, most recently, back to follicular carcinoma.

## INTRODUCTION

Papillary thyroid carcinoma (PTC) is a nuclear diagnosis. This is what most pathologists were taught as trainees, and those of us familiar with thyroid histopathology and cytopathology did not think twice about the implications of this statement. However, others who are unfamiliar with this concept may be confused about the relationship between papillary (a cellular architectural term) and nuclear features. So, what are the nuclear features of PTC and why are they controversial? The history of PTC nuclei has not been straightforward, and its path has led to two main morphologies with different biologic characteristics. Our evolving understanding of PTC nuclei has influenced histopathologic and cytopathologic classification systems. Although appending specific names to the two main morphologic archetypes of PTC nuclei gives the impression that these entities are morphologically discrete, in reality, PTC nuclear changes are present in a continuum. Thus, differing opinions regarding gray zone interpretations have resulted in controversies, interobserver variability, and debate. More recently, the advent of molecular testing on thyroid fine needle aspiration specimens has shed light on additional layers of objective information to enhance our understanding of the two similar yet biologically distinct nuclei. In this review, we summarize the major events representing the paradigm shifts, classification system changes, and controversies that developed our current understanding (Table 1).

**TABLE 1:** Key historical publications regarding nuclear and other pathologic features of PTC.

References	Architectural/nuclear description	Comment
Wilson 1921^1^	Malignant neoplasms noted for increased nuclear size.	Malignant diagnosis based on presence of structural invasion or metastasis. Any sizable adenoma in active proliferation was considered potentially malignant, even if encapsulated.
Warren and Adler 1958^2^ Woolner 1961^3^	Papillary architecture in neoplasms became an indicator of malignancy, regardless of invasion status. Neoplasms with mixed papillary and follicular patterns behaved like PTC.	Papillary carcinoma often demonstrated lymphatic invasion and/or lymph node metastasis, but rare distant metastasis.
Lindsay 1960^4^	Exquisite description of PTC nuclei, characterized as having ground‐glass appearance.	FVPTC is born; however, it was classified under FC and was not yet commonly recognized. Landmark paper recognizing the importance of nuclear features in PTC.
Gray and Doniach 1968^5^	By electron microscopy, Gray and Doniach described pseudoinclusion as invaginated cytoplasmic material enclosed by a membrane within the nucleus.	While the presence of pseudoinclusions was not specific to any one entity, this feature became one of the integral nuclear criteria of PTC.
Chen and Rosai 1977^6^	Round or oval Optically clear or ground glass Scant amount of finely dispersed chromatin Small indistinct nucleolus Single, large, oval, or round clear space	Revival of FVPTC (now as a PTC subtype). The 6 FVPTC cases described were not subtle; all were infiltrative, 5/6 with moderate to marked fibrosis, 3 had no capsule, and 3 had partial encapsulation (by current criteria, they would be categorized as at least infiltrative FVPTC).
LiVolsi and Asa 1994^7^	Nuclei in a large area of follicular lesion Enlarged nuclei Ovoid Cleared chromatin Grooves	Nuclear criteria for FVPTC expanded to include cases with subtle nuclear and histologic features. Increased incidence of FVPTC diagnoses corresponded with decrease in FC diagnoses.
Hirokawa 2002^8^	Subjectivity in the interpretation of nuclear clearing was one of the main factors influencing interobserver variability regarding the diagnosis of PTC.	Interobserver variability study on encapsulated thyroid follicular lesions among 4 American and 4 Japanese pathologists demonstrated complete agreement among all experts in only 10% of cases.
Lloyd 2004^9^	Nuclear pseudoinclusions, grooves, and ground glass chromatin stated as major criteria for the diagnosis of FVPTC.	Interobserver variability study on FVPTC. Complete concordance on FVPTC diagnosis was achieved in 39% of cases. For FVPTC cases with metastasis, 66.7% had complete concordance. Application of criteria varied and was subjective among experts.
Liu 2006^10^	FVPTC categorized into 2 main groups Infiltrative subtype: similar to conventional PTC Encapsulated subtype: similar to FA/FC with papillary nuclear features diffuse in 87% and multifocal in 13%.	Encapsulated noninvasive FVPTC was identified as an indolent disease with questionable malignant potential.
El Shiekh 2008^11^	Nuclear clearing, grooves, overlapping, crowding, membrane irregularity, and enlargement were the most helpful features in making diagnosis of FVPTC.	Experts unanimously agreed on the FVPTC diagnosis in only 13% of cases.
Nikiforov 2016^12^	NIFTP “papillary‐like” nuclear features Nuclear grading based on Size and shape Membrane irregularities Chromatin characteristics	Semiquantitative criteria and example images provided minimum standards for acceptance for papillary‐like nuclear features (nuclear score 2).
Thompson 2018^13^	International interobserver variability on nuclear scoring system for NIFTP	Good to substantial interobserver agreement among pathologists from the USA, Japan, and UK.
Jung 2021^14^	1. Nuclear enlargement 2. Nuclear crowding/overlapping 3. Nuclear elongation 4. Irregular nuclear membranes 5. Nuclear grooves 6. Chromatin clearing 7. Sickle‐shaped nuclei 8. Nuclear pseudoinclusions	Each parameter was graded and cases with *BRAF V600E* or *BRAF V600E‐*like mutations had nuclear pseudoinclusions and significantly higher nuclear scores than those with *RAS* or *RAS‐*like mutations.
Kakudo 2022^15^	“Delicate nuclear features” of *RAS*‐like follicular patterned thyroid tumors, also known as PTC‐type nuclear features or “papillary‐like nuclear features” by NIFTP working group, were characterized by: Nuclear enlargement Membrane irregularity Chromatin clearing These contrasted to “fully developed PTC‐type nuclear changes” that were associated with *BRAF V600E* or *BRAF V600E‐*like mutations.	Asian pathologists often classified “delicate nuclear features” in follicular patterned thyroid tumors as FA or FC, whereas Western pathologists categorized the similar tumors as FVPTC.
Harahap 2025^16^	Features associated with *BRAF V600E* or *BRAF V600E‐*like mutations 1. Nuclear elongation 2. Nuclear grooves 3. Sickle‐shaped nuclei 4. Nuclear pseudoinclusions	Nuclei associated with *BRAF V600E* or *BRAF V600E‐*like mutations showed florid nuclear features and high nuclear score compared to nuclei associated with *RAS* or *RAS‐*like mutations which showed subtle features.
Williams 2025^17^	Revisited NIFTP “papillary‐like” nuclear features	World‐wide adoption rate of NIFTP is high. However, Western pathologists had a tendency to decrease their thresholds for papillary nuclei compared to their counterparts in Asia and Oceania.

Abbreviations: FA, follicular adenoma; FC, follicular carcinoma; FVPTC, follicular‐variant papillary thyroid carcinoma; NIFTP, noninvasive follicular thyroid neoplasm with papillary‐like nuclear features; PTC, papillary thyroid carcinoma.

### Early classification dilemmas: before the appreciation of PTC nuclear features

In the early part of the 20th century, pathologic diagnosis of thyroid malignancies was considered challenging because of the great variation in histomorphologic changes and their resemblance to nonmalignant counterparts. The distinction between benign and malignant neoplasms was made by the presence or absence of structural invasion or metastasis. In his paper on malignant thyroid tumors, Louis B. Wilson of the Mayo Clinic postulated that “malignant adenoma” (classified today as follicular carcinoma [FC]) represented a precursor of “malignant papilloma” (classified today as PTC), perhaps because the former was often encapsulated and the latter infiltrative in growth pattern.^1^ Some neoplasms, showing a combination of follicular and papillary growth patterns, were thought to represent an intermediary step. Interestingly, Wilson mentioned that any sizable adenoma in active proliferation is potentially malignant, even if encapsulated. Descriptions on nuclear details were relatively sparse and limited to size (small or large) and staining intensity.

By the middle of the 20th century, the classification of malignant thyroid neoplasms was becoming codified by architectural patterns into FC, PTC, medullary thyroid carcinoma, and anaplastic thyroid carcinoma.^2^, ^18^ Although histologic invasion definitely would establish the diagnosis of malignancy, papillary architectural pattern was drawing attention in the 1950s and 1960s because papillary neoplasms, even when noninvasive, were reported to develop metastasis.^19^, ^20^ Therefore, while a follicular‐patterned neoplasm may be an adenoma or carcinoma, papillary architecture in a neoplasm became an indicator of malignancy, regardless of invasion status. One of the dilemmas arising from the period was the classification of neoplasms with both follicular and papillary patterns. Were these tumors a combination of two phenotypically different neoplasms, one neoplasm showing divergent patterns, or progression of one phenotype to another? Later, Woolner et al. demonstrated that neoplasms with mixed papillary and follicular patterns behaved like PTC, even when the papillary component was minor.^3^

### PTC becomes a nuclear diagnosis

In the second half of the 20th century, nuclear features in thyroid neoplasms were increasingly recognized by their roles in disease classification. Descriptions of follicular‐patterned neoplasm with features known today as papillary nucleus were made by Crile and Hazard in 1953 and proposed as “alveolar variant of papillary carcinoma.”^21^ However, the landmark paper by Lindsay in 1960 brought attention to the nuclear details of PTC.^4^ He exquisitely described the nuclear features, focusing on the nuclear membranes and chromatin pattern. The nuclear chromatin was characterized as “opaque with large segments devoid of chromatin and as though it was composed of ground glass.” Nucleoli were small in most nuclei, although some were large, discrete, and eosinophilic. The nuclear membranes frequently had folds and indentations when observed under phase microscopy. Mitoses were infrequent. These nuclear features were observed not only in conventional PTC, but also in the follicular variant of papillary thyroid carcinoma (FVPTC), an entity first coined by Lindsay but classified as a subtype under FC in his publication. Later in the same decade, Gray and Doniach added the presence of pseudoinclusions as an important feature of PTC.^5^ By electron microscopy, they demonstrated that these pseudoinclusions were invaginated cytoplasm enclosed by a membrane, hence not true inclusions. In the ensuing decades, these “classical papillary” nuclear features became integral to the diagnosis of PTC. One of the most striking features of PTC was the “empty” appearance most readily seen on histologic sections. This feature was initially dubbed “hot eyes” by Nathan Friedman (based on their similarity in his mind to those of the American actress Martha Scott) and later renamed “Orphan Annie eyes” by Nancy Warner, who adapted the appearance from a popular 20th‐century comic strip character.^22^, ^23^ Initially, the nuclear clearing known as Orphan Annie eyes was thought to represent formalin fixation artifact; however, subsequent studies suggested that chromatin change alone or the combination of chromatin change and perinuclear changes contributed to the ground‐glass nuclear changes.^24^, ^25^ Later in the 20th century, four nuclear characteristics, nuclear enlargement, ground glass chromatin, pseudoinclusions, and grooves, became recognized as key diagnostic features of PTC.^26^ However, as more PTC variants were recognized, the degree to which each nuclear feature was required to make the diagnosis of PTC became somewhat subjective and varied from pathologist to pathologist.

### Evolution of FVPTC and the expanded interpretation of PTC nuclei

Although Lindsay coined the term FVPTC in 1960, this term did not enter most pathologists’ lexicon until the latter part of the 20th century. In their 1977 report, Chen and Rosai provided details on six thyroid neoplasms composed of follicles of varying sizes and no overt papillary structures.^6^ All six cases had infiltrative borders, with three showing no encapsulation and the remaining three exhibiting partial encapsulation. The nuclei were round or oval with ground‐glass appearance and scant finely dispersed chromatin. Many of the nuclei demonstrated a single, large, oval or round clear space. Interestingly, four of five cases with metastasis showed mixed follicular and papillary patterns in regional lymph nodes. This report revived the FVPTC terminology and aligned this neoplasm with PTC rather than FC (in contrast to what Lindsay had in his 1960 publication). By current diagnostic standards, Chen and Rosai’s cases would be categorized likely as infiltrative FVPTC (which is akin in behavior to conventional PTC and distinct from encapsulated FVPTC with invasion) or even conventional PTC with prominent follicular pattern.

As PTC evolved from an architectural to a nuclear diagnosis, debate and contention surrounding the nuclear thresholds for making the PTC diagnosis ensued in the late 20th century and early 21st century. In North America, subtle nuclear changes became acceptable for PTC and, consequently, more follicular‐patterned neoplasms (even those that were encapsulated and not infiltrative) migrated from follicular adenoma (FA) and FC to FVPTC.^27^, ^28^, ^29^ Consequently, its incidence surpassed that of conventional PTC and FC in some institutions.^7^ However, the minimal threshold features acceptable for the diagnosis of PTC (e.g., nuclear enlargement, chromatin changes) were difficult to define and were subject to interpretation. This practice pattern may have been influenced, in part, by the medical‐legal climate that was concerned about false‐negative diagnoses (e.g., cervical‐vaginal Pap smear practice) of this era.^30^, ^31^ In these regions, making a malignant diagnosis for cases with subtle features may have been considered a “safer” diagnosis, if recurrence or metastasis was observed years later. Reports of partially or completely encapsulated FVPTC (some that were initially diagnosed as FA) with development of bone metastasis heightened the sensitivity for this diagnosis.^32^ However, the expanded and more comprehensive interpretation of the nuclear features was not accepted by all individuals and institutions, particularly in Asia, some parts of North America, and other parts of the world.^33^, ^34^ Questions emerged as to whether the minimal criteria such as nuclear enlargement and ground glass chromatin were sufficient for PTC diagnosis or too sensitive and lacking in specificity.

By the early 2000s, the worldwide recognition of interobserver variability in PTC interpretation led to a number of studies comparing the diagnostic patterns of thyroid pathology experts.^8^, ^9^, ^11^ The international panel of experts agreed that nuclear pseudoinclusions, grooves, and ground‐glass chromatin were the major criteria to diagnose PTC; however, the weight placed on each of these as well as other factors varied considerably, and unanimous agreement was reached in only a minority of cases. As a result, these international interobserver variability studies uncovered two types of nuclei, “classical papillary nucleus” with robust features including nuclear enlargement, elongation, ground‐glass chromatin, grooves, and intranuclear pseudoinclusions and “papillary‐like nucleus” with subtle nuclear features, especially in follicular‐patterned nodules. Although the classical papillary nucleus was accepted widely, the papillary‐like nucleus demonstrated low interobserver agreement. The lack of consensus for minimum nuclear criteria for the diagnosis of FVPTC was acknowledged in this era and individual practices continued to diverge in the interpretation of the nucleus associated with PTC.^35^, ^36^ Dr. Austin Vickery made an insightful statement in the late 20th century regarding the differential diagnosis of FA and PTC, “some criteria may be indefinite and vulnerable to subjective morphological interpretation.”^37^ The interpretive problem regarding the less well‐developed nuclei, especially for encapsulated well‐differentiated follicular‐patterned thyroid nodules, became increasingly apparent in the latter part of the first decade of the 21st century. In 2008, Dr. Juan Rosai mentioned that those lesions constituted the majority of his consultation practice.^36^

Although rare instances of partially or completely encapsulated FVPTC developing distant metastasis were reported previously, the early 2000s also brought concern regarding the indolent nature of encapsulated well‐differentiated follicular‐patterned thyroid nodules and the overdiagnosis of FVPTC in North America. Retrospective follow‐up studies on encapsulated noninvasive FVPTCs revealed very low incidence of recurrence and cancer‐related deaths.^27^, ^28^, ^29^ In 2006, Liu et al. studied 78 cases of FVPTCs that were encapsulated (*n* = 61) or infiltrative (*n* = 17).^10^ They found that infiltrative FVPTCs behaved like conventional PTC with invasion into the surrounding tissue, intratumoral fibrosis, extrathyroidal extension, and more frequent positive resection margins, whereas encapsulated FVPTCs behaved like FA or FC. None of the noninvasive, encapsulated FVPTCs developed recurrence. Thus, in contrast to the safer approach to guard against metastases, the counternarrative that “labeling such tumors as ‘cancer’ is actually more harmful to patients” began to surface. In 2012, the National Cancer Institute met to address the problem of “overdiagnosis,” releasing a statement that included consideration to rename indolent lesions at various organ sites, including thyroid.^38^

### Molecular testing clarifies the dividing line between the two PTC nuclei (classical papillary and papillary‐like)

By adding molecular objectivity, Rivera et al. demonstrated that encapsulated FVPTCs had a high rate of *RAS* mutations and absent *BRAF V600E* mutations, whereas infiltrative FVPTCs had molecular profiles similar to those of conventional PTC with more frequent *BRAF V600E* mutations.^39^ By 2014, molecular analysis of nearly 500 PTCs published by The Cancer Genome Atlas Research Network identified two main drivers of PTC with fundamentally different biological characteristics.^40^ First, PTCs with *BRAF V600E* mutations were associated mostly with conventional or tall‐cell subtype PTC. The Cancer Genome Atlas also uncovered *BRAF V600E*–like mutations (which were not *BRAF V600E* mutations yet demonstrated similar expressions, signaling, and behavioral characteristics) that had similar outcome associations. Second, PTCs with *RAS* mutations were predominantly FVPTC. Analogously, *RAS*‐like mutations (which did not involve *RAS* mutation yet demonstrated similar expression, signaling, and behavioral characteristics) also were associated with FVPTC. However, *RAS* and *RAS*‐like mutations were not specific for FVPTC and were found in other follicular‐patterned neoplasms (e.g., FA, FC).^41^ Consequently, the increasing application of molecular testing platforms on thyroid fine needle aspiration specimens, gave investigators and practitioners better distinction between the subtypes of PTCs, with insights beyond what can be attained by morphology‐based pathology alone.^42^ During the middle of the 2010s, the need to revise the nomenclature for noninvasive, encapsulated FVPTCs to a nonmalignant term was recognized. This movement culminated in the establishment of noninvasive follicular thyroid neoplasm with papillary‐like nuclear features (NIFTP) in 2016.^12^ Although NIFTP was intended to convey a nonmalignant entity (because the probability of metastasis and morbidity was exceedingly low), it was not entirely benign either since it was associated with *RAS* or *RAS*‐like mutations and had the potential for progression with acquisition of additional genomic alterations. Also noted was the inclusion of the term “papillary‐like nuclear features” in NIFTP (rather than “papillary nuclear features”).

**FIGURE 1:**
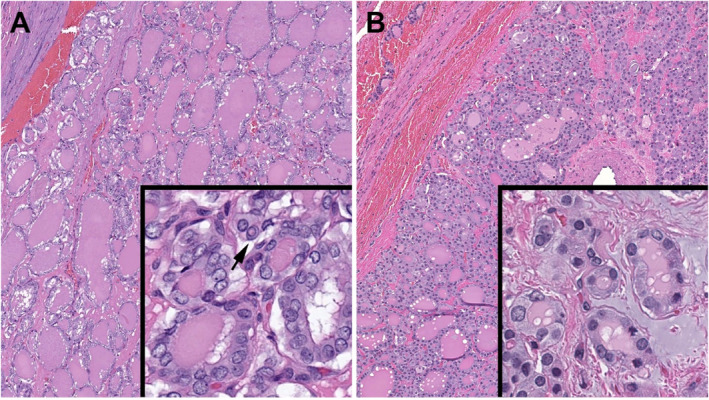
Comparison of “classical papillary nucleus” to “papillary‐like nuclei” in neoplastic thyroid follicles. (A) The robust papillary nuclear features with nuclear enlargement and elongation, membrane irregularities with pseudoinclusions (arrow), and chromatin clearing are uniformly accepted as diagnostic of PTC. This neoplasm was diagnosed as conventional PTC with *BRAF V600E* mutation and lymph node metastasis (hematoxylin and eosin stain). (B) In contrast, the subtle nuclear features are accepted variably as to whether the changes are interpreted as “papillary‐like nuclear features” or not. Also note the range of changes from nucleus to nucleus in this case of NIFTP with *RAS* mutation (hematoxylin and eosin stain). NIFTP indicates noninvasive follicular thyroid neoplasm with papillary‐like nuclear features; PTC, papillary thyroid carcinoma.

**FIGURE 2:**
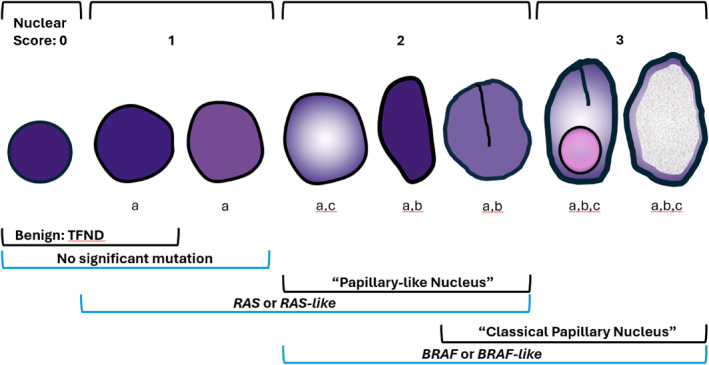
Diagrammatic illustration of thyroid nuclear features. Nuclear score is based on (A) nuclear enlargement and/or elongation, (B) membrane irregularities including grooves and pseudoinclusions, and (C) chromatin characteristics such as clearing and ground glass appearance. The typical morphologic nuclear representation for the type of nucleus (e.g., benign: thyroid follicular nodular disease [TFND], papillary‐like nucleus) are enclosed within the black bracket. However, genomic features (e.g., no significant mutation, *RAS or RAS*‐like) extend beyond morphologic changes and are enclosed within the blue bracket. Therefore, overlap exists in the genomic alterations for the various morphologic changes.

In addition to the nomenclature revision, the inception of NIFTP made the thyroid pathologists take a closer look at the criteria for the “papillary‐like nucleus” and its reproducibility, since its interpretation had been subjective and not uniformly accepted, as discussed previously. To provide objective guidelines, a nuclear scoring scheme (including nuclear size and shape, membrane irregularities, and chromatin characteristics) was established.^12^, ^43^ By this nuclear scoring scheme, a minimum score of two of three was required for NIFTP. Subsequent histologic‐molecular correlation studies demonstrated that thyroid neoplasms with *BRAF V600E* or *BRAF V600E*–like mutations were associated significantly with high nuclear scores and intranuclear pseudoinclusions when compared to neoplasms with *RAS* or *RAS*‐like mutations.^14^, ^16^ Therefore, “classical papillary nucleus” often correlated with papillary architecture, *BRAF V600E* or *BRAF V600E*–like mutations, and corresponding clinical features, whereas subtle and delicate papillary‐like nucleus often correlated with follicular architecture and *RAS* or *RAS*‐like mutations (Table 2). However, notable exceptions were infiltrative FVPTC, a predominantly follicular‐patterned (with focal papillary architecture) neoplasm with *BRAF V600E* or *BRAF V600E*–like mutations and noninvasive encapsulated papillary RAS‐like thyroid tumor with *RAS* or *RAS*‐like mutations.^39^, ^44^ Therefore, the pathobiologic division of differentiated thyroid neoplasms became more accurately classified according to *BRAF* vs *RAS* alterations than papillary vs follicular histologic architecture. (Figure 1, Figure 2) Although an earlier international interobserver variability study demonstrated that the NIFTP nuclear scoring system had good‐to‐substantial interobserver agreement, a more recent survey of an international group of pathologists found that, despite a high adoption rate of NIFTP globally, there still was a significant difference in the threshold for making the diagnosis of NIFTP across Eastern and Western pathology practices.^13^, ^17^ Eastern (Asian) practices tended to place neoplasms with subtle and delicate nuclear features (papillary‐like nucleus) into the FA–FC spectrum, whereas Western (North American and European) practices tended to place these neoplasms in the NIFTP–FVPTC spectrum. Consequently, the reported incidence of *BRAF V600E* or *BRAF V600E*–like mutations in PTC, which is high (50%–90%) in Eastern practices and low (35%–50%) in Western practices was brought to question and may reflect differences in how pathologists classify these neoplasms rather than inherent genomic differences in the populations.^15^

**TABLE 2:** Associations of classical papillary nucleus and papillary‐like nucleus in thyroid neoplasms.

Features	Classical papillary nucleus	Papillary‐like nucleus
Molecular associations	*BRAF V600E* or *BRAF V600E*‐like	*RAS* or *RAS*‐like
Typical nuclear features (working definitions)	Enlarged and often elongated Nuclear grooves Pseudoinclusions Chromatin clearing, ground glass features Higher nuclear scores	Mildly enlarged and round Slightly or subtly irregular nuclear membranes Mild nuclear chromatin clearing Often lacks pseudoinclusions Lower nuclear scores
Histologic architectural/cellular pattern associations	Papillary Solid Tall cell Hobnail Follicular (infiltrative FVPTC)	Follicular Trabecular
Gross/imaging associations	Infiltrative, taller than wide	Well‐circumscribed, noninfiltrative
Cytology TBSRTC associations	Malignant, SFM, AUS‐NA	FN, AUS‐O
Histopathologic diagnostic associations	Conventional PTC Tall cell PTC Columnar PTC Hobnail PTC Infiltrative FVPTC	FA NIFTP FC Encapsulated FVPTC

Abbreviations: AUS‐NA, atypia of undetermined significance‐nuclear atypia; AUS‐O, atypia of undetermined significance‐other; FA, follicular adenoma; FC, follicular carcinoma; FN, follicular neoplasm; FVPTC, follicular‐variant papillary thyroid carcinoma; NIFTP, noninvasive follicular thyroid neoplasm with papillary‐like nuclear features; PTC, papillary thyroid carcinoma; SFM, suspicious for malignancy; TBSRTC, the Bethesda System for Reporting Thyroid Cytopathology.

**FIGURE 3:**
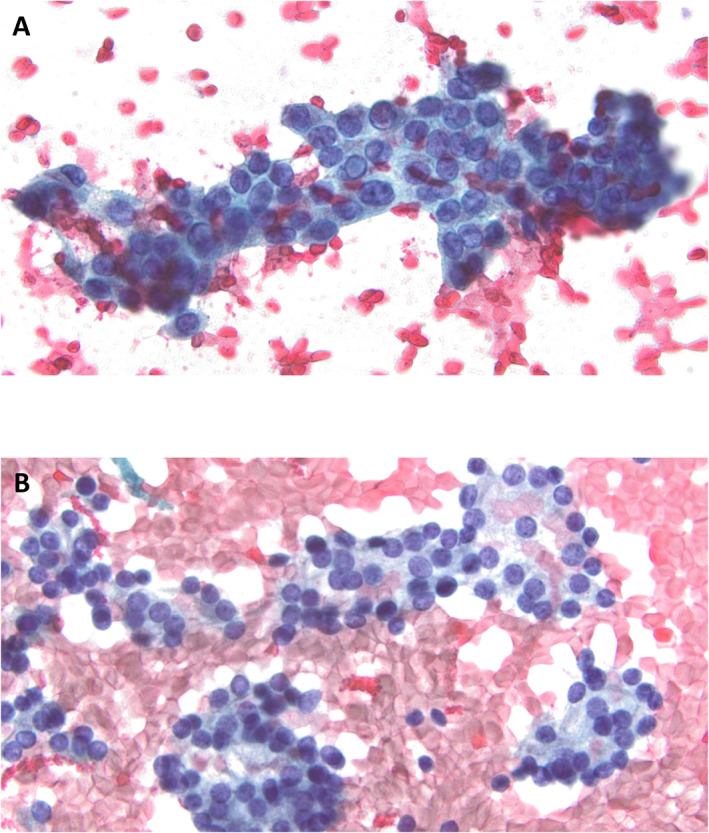
Comparison of classical papillary nucleus to papillary‐like nuclei in cytology specimens. (A) Robust nuclear features including pronounced nuclear enlargement, irregular nuclear membranes, groove formation, and pseudoinclusions are readily noted in this case of PTC with *BRAF V600E* mutation (Pap stain). (B) Cytologic case of FN with *RAS* mutation and eventual resection diagnosis of FVPTC shows mild nuclear enlargement with occasional membrane irregularities, and slight chromatin clearing (Pap stain). FN indicates follicular neoplasm; FVPTC, follicular variant papillary thyroid carcinoma; PTC, papillary thyroid carcinoma.

In parallel to histologic samples, cytology specimens also demonstrate nuclear changes along *BRAF V600E* and *RAS* lines. However, some notable differences between histologic and cytologic preparations exist. Although nuclear membrane irregularities, chromatin clearing, grooves, and pseudoinclusions are observed in both preparations, some nuclear features of PTC on cytology preparations (e.g., Orphan Annie nuclei) may not be as apparent (Figure 3). Because changes of PTC may be focally present within a nodule (sprinkling sign), cytology sampling may not capture the most diagnostic areas. Nonetheless, since genomic alterations often precede morphologic changes, molecular testing on cytology samples has the potential to reveal the underlying biologic characteristics in cases with subtle cytologic changes. Although neoplasms with *BRAF V600E* or *BRAF V600E*–like mutations are diagnosed typically as suspicious for malignancy (SFM) or malignant, neoplasms with *RAS* or *RAS‐*like mutations often are given indeterminate diagnoses: atypia of undetermined significance (AUS), follicular neoplasm (FN), or less commonly SFM.^45^ Although the cytologic division between *BRAF V600E* and *RAS* tumors may appear straightforward, the historic inception of NIFTP in 2016 resulted in a shift in cytologic thresholds for the interpretation of nuclear features. Development of cytologic nuclear grading schemes comparable to the NIFTP nuclear scoring scheme addressed the cytologic nuclear features of follicular‐patterned neoplasms and revealed that cytomorphology could not distinguish NIFTP from FVPTC and that neoplasms that were reclassified as NIFTP occasionally had malignant presurgical cytology diagnoses.^46^, ^47^, ^48^ Because a malignant cytology diagnosis would be considered an overcall by current standards, the threshold for the malignant cytology diagnosis was raised in the second edition of the Bethesda System for Reporting Thyroid Cytopathology in 2017 to avoid false‐positive diagnoses for eventual NIFTP cases.^49^ Cytology cases not reaching the new threshold for the malignant cytopathology diagnosis were recommended to be classified as AUS with nuclear atypia or SFM.^50^ Furthermore, follicular‐patterned cytology cases with nuclear features bordering on SFM were advised to be diagnosed as FN to avoid overtreatment. Subsequent review of the literature also substantiated the inability of cytologic features to distinguish between NIFTP and FVPTC.^51^

### Remaining controversies and open questions

**FIGURE 4:**
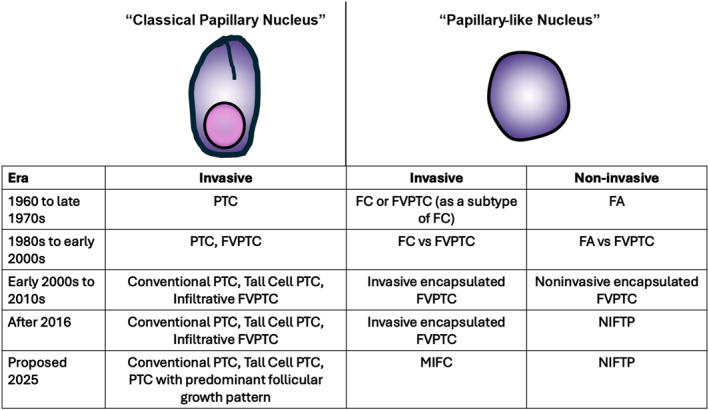
Diagnostic associations of classical papillary nucleus and papillary‐like nucleus over the past several decades. The classical papillary nucleus has been associated with conventional PTC, tall‐cell PTC, and infiltrative FVPTC, which recently has been proposed to be renamed PTC with predominant follicular growth pattern.^52^ The papillary‐like nucleus with subtle nuclear features became increasingly associated with FVPTC after the 1980s. Because of the indolent behavior, noninvasive encapsulated neoplasm with papillary‐like nucleus was later renamed NIFTP. On the other hand, invasive encapsulated FVPTC has been proposed to be included in MIFC.^52^ FA indicates follicular adenoma; FC, follicular carcinoma; FVPTC, follicular‐variant papillary thyroid carcinoma; MIFC, minimally invasive follicular carcinoma; NIFTP, noninvasive follicular thyroid neoplasm with papillary‐like nuclear features; PTC, papillary thyroid carcinoma.

While worldwide communication and international efforts by groups such as the World Health Organization have enhanced consensus on disease and tumor classification, controversies remain regarding the PTC nuclei. The well‐developed classical papillary nucleus is associated genotypically with *BRAF V600E* or *BRAF V600E*–like mutations and phenotypically with conventional PTC, tall‐cell PTC, and other PTCs with robust nuclear features. There is little controversy surrounding these morphologic changes and neoplasms. In contrast, subtle and delicate papillary‐like nucleus is associated genotypically with *RAS* or *RAS*–like mutations and phenotypically with follicular‐patterned neoplasms. As stated previously, Western practices have an increased tendency to diagnose tumors with *RAS* or *RAS*‐like mutations into the NIFTP–FVPTC spectrum, whereas Eastern practices place similar tumors into the FA–FC spectrum. Historically, debates focused on whether noninvasive encapsulated neoplasms with borderline papillary‐like nucleus should be classified as FA or NIFTP and similar invasive encapsulated neoplasms, FC or FVPTC. The difference in the interpretation of the nuclear features may persist in different parts of the world for the foreseeable future. However, given our current understanding that these neoplasms are likely associated with *RAS* or *RAS*‐like mutations, the debate between FA/FC versus NIFTP/FVPTC has become less critical because the biologic behavior and management approaches are similar. In this context, Asa et al. have proposed eliminating the terminology of invasive encapsulated FVPTC and including this neoplasm under minimally invasive FC.^52^ The story has come full circle (Figure 4). In 1960, Lindsay classified FVPTC under FC and, later, it was categorized as a subtype of PTC. The recent proposal supports Lindsay’s original concept and places FVPTC back with FC. Questions remain regarding the threshold for the recognition of the papillary‐like nucleus and whether the papillary‐like nucleus should remain a PTC‐associated feature or included in the broader spectrum of FA/FC.

## CONCLUSIONS

The nucleus associated with PTC has had a fascinating history. We have witnessed discoveries and debates regarding the nuclear changes associated with the diagnosis of PTC. Two main morphologic archetypes of PTC nuclei, classical papillary nucleus and papillary‐like nucleus, are recognized and are associated with *BRAF V600E* or *BRAF V600E*–like mutations and *RAS* or *RAS*‐like mutations, respectively. Cytologically, classical papillary nucleus often is associated with overt nuclear features of PTC (e.g., nuclear enlargement, elongation, grooves, pseudoinclusions, chromatin clearing), papillary growth pattern, and Bethesda Thyroid Cytology diagnoses of malignant, SFM (with features just short of a malignant diagnosis) or AUS‐nuclear atypia (with concerning features less completely developed). In contrast, “papillary‐like nucleus” typically demonstrates more subtle features (e.g., mild enlargement, retention of round shape, lack of pseudoinclusions) and is associated with FN or AUS‐other cytology diagnoses. Although these descriptions give the impression that these entities are morphologically discrete, in reality, these changes are present in a continuum and interobserver variability is recognized, both nationally and internationally. In this regard, molecular testing can assist in clarifying the pathobiologic nature of the nodule, when the morphologic features are subtle or present focally. The proposed terminology change of eliminating the use of invasive encapsulated FVPTC and including this neoplasm under minimally invasive FC may reduce semantic debate. Nonetheless, awareness of classical papillary nucleus and papillary‐like nucleus in surgical pathology and cytopathology specimens remains important because of their molecular associations and prognostic implications.

## CONFLICT OF INTEREST STATEMENT

Yuri E. Nikiforov owns intellectual property related to ThyroSeq and receives royalty; he serves as a consultant for Sonic Healthcare USA. The remaining authors have no conflict of interest.
